# Evidence of selection as a cause for racial disparities in fibroproliferative disease

**DOI:** 10.1371/journal.pone.0182791

**Published:** 2017-08-08

**Authors:** Jacklyn N. Hellwege, Eric S. Torstenson, Shirley B. Russell, Todd L. Edwards, Digna R. Velez Edwards

**Affiliations:** 1 Division of Epidemiology, Department of Medicine, Vanderbilt University Medical Center, Nashville, TN, United States of America; 2 Vanderbilt Genetics Institute, Vanderbilt University Medical Center, Nashville, TN, United States of America; 3 Department of Obstetrics and Gynecology, Vanderbilt University Medical Center, Nashville, TN, United States of America; Ospedale San Pietro Fatebenefratelli, ITALY

## Abstract

Fibroproliferative diseases are common complex traits featuring scarring and overgrowth of connective tissue which vary widely in presentation because they affect many organ systems. Most fibroproliferative diseases are more prevalent in African-derived populations than in European populations, leading to pronounced health disparities. It is hypothesized that the increased prevalence of these diseases in African-derived populations is due to selection for pro-fibrotic alleles that are protective against helminth infections. We constructed a genetic risk score (GRS) of fibroproliferative disease risk-increasing alleles using 147 linkage disequilibrium-pruned variants identified through genome-wide association studies of seven fibroproliferative diseases with large African-European prevalence disparities. A comparison of the fibroproliferative disease GRS between 1000 Genomes Phase 3 populations detected a higher mean GRS in AFR (mean = 148 risk alleles) than EUR (mean = 136 risk alleles; T-test p-value = 1.75x10^-123^). To test whether differences in GRS burden are systematic and may be due to selection, we employed the quantitative trait loci (QTL) sign test. The QTL sign test result indicates that population differences in risk-increasing allele burdens at these fibroproliferative disease variants are systematic and support a model featuring selective pressure (p-value = 0.011). These observations were replicated in an independent sample and were more statistically significant (T-test p-value = 7.26x10^-237^, sign test p-value = 0.015). This evidence supports the role of selective pressure acting to increase frequency of fibroproliferative alleles in populations of African relative to European ancestry populations.

## Introduction

Fibroproliferative diseases are a consequence of dysregulated scarring and connective tissue overgrowth, affect many organ systems, vary widely in presentation, and are very common in humans[1, 2]. Uterine fibroids, keloid scars, pulmonary fibrosis, cirrhosis, Crohn’s disease, and atherosclerosis are examples of diseases with fibroproliferative features. Many fibroproliferative diseases are more prevalent in recently African-derived populations than in European populations[3], collectively contributing to pronounced overall health disparity (Table 1). For example, keloids are more common in those with darker pigmentation[4], and systemic scleroderma[5, 6], nephrosclerosis[7], and sarcoidosis[8] are more prevalent in African American individuals. However, this is not the case for all fibroproliferative diseases. Dupuytren contracture is a disease predominantly affecting European American individuals[9], as are pulmonary fibrosis[10] and multiple sclerosis[11], though they are not uncommon in those of recent African ancestry.

**Table 1:** Fibroproliferative diseases with increased prevalence in African-derived populations.

Disease	Prevalence Ratio (AA:EA)	Number of SNPs included in GRS^1^
Nephrosclerosis	3–20[35–41]	69
Keloids	20[42]	3
Sarcoidosis	3–17[43–51]	1
Hypertension^2^	1.4–7[52–57]	42
Glaucoma	4–5[58, 59]	21
Scleroderma	3[43, 60–62]	8
Uterine Fibroids	1.5–3[63–66]	3

Table modified from Russell et al, 2015[3]. AA-African American; EA-European American

^1^Number of loci reaching genome-wide significance in the NHGRI/EBI GWAS Catalog (www.ebi.ac.uk/gwas). Complete list of SNPs is contained in S1 Table

^2^Hypertension is associated with a systemic inflammatory state which can lead to target organ fibrosis[67–70], and also occurs in the presence of atherosclerosis which is itself fibroproliferative[71–73]. There are shared characteristics between hypertension and other fibroproliferative diseases[54, 74–78], and fibrotic growth factors are often upregulated in hypertension as well[53, 55, 79, 80]. Hypertension has been presented as a fibroproliferative condition in other publications[1, 3, 81], which discuss the evidence for this in more detail.

Beyond the large prevalence disparities across continental ancestral groups, there is observational association study evidence to suggest that these conditions are heritable[12–17]. Genome-wide association studies (GWAS) have identified many common susceptibility variants for several fibroproliferative diseases[18, 19]. However, like most phenotypes, these studies have been performed predominantly in European American populations.

It has been suggested that fibroproliferative diseases may share a common genetic background[20]. There is also evidence for pathological similarities across fibroproliferative phenotypes[1, 2]. A recent review by Russell et al presents the hypothesis and presents evidence for the increased prevalence of fibroproliferative diseases in African American populations as a result of selection for anti-helminthic, pro-fibrotic alleles in response to helminth infections on the African continent[3]. Similar scenarios of diseases arising due to selective response to pathogens in African populations have been seen in sickle cell disease conferring resistance to malaria[21, 22], and the increased frequency of chronic kidney disease in carriers of Apolipoprotein L1 (*APOL1*) variants, which offer enhanced ability to resist trypanosome infections that cause African sleeping sickness[23].

When a trait is under adaptive selective pressure, allele frequencies at all loci with an influence on that trait will change over generations. In contrast, under neutrality allele frequencies will drift randomly. Thereby, if individuals with higher values for the trait enjoy higher relative fitness, then trait-increasing alleles will tend to become more common over generations, and the effect of selection on allele frequency will be proportional to the effect of the allele on the trait under selection. For a complex trait under selection with many genetic determinants with subtle effects, small changes in allele frequency will occur with relatively undetectable differences in linkage disequilibrium (LD) and haplotype diversity, as has been seen in human height[24]. Across trait loci, systematic differences in trait- or risk-increasing allele frequencies that are consistent with the disparity between two populations are detectable, given a sufficient number of known causal loci and precision to estimate allele frequencies[25, 26]. In this study, we were able to assess systematic frequency differences of known fibroproliferative risk-increasing alleles across populations; however, we could not assume or assess proportionality of effect sizes to allele frequency differences between African and other continental populations. The available effect size estimates for these alleles are not for their putative protective effects on helminth infections, but for their consequences across organ systems in various fibroproliferative traits.

## Results and discussion

**Fig 1:**
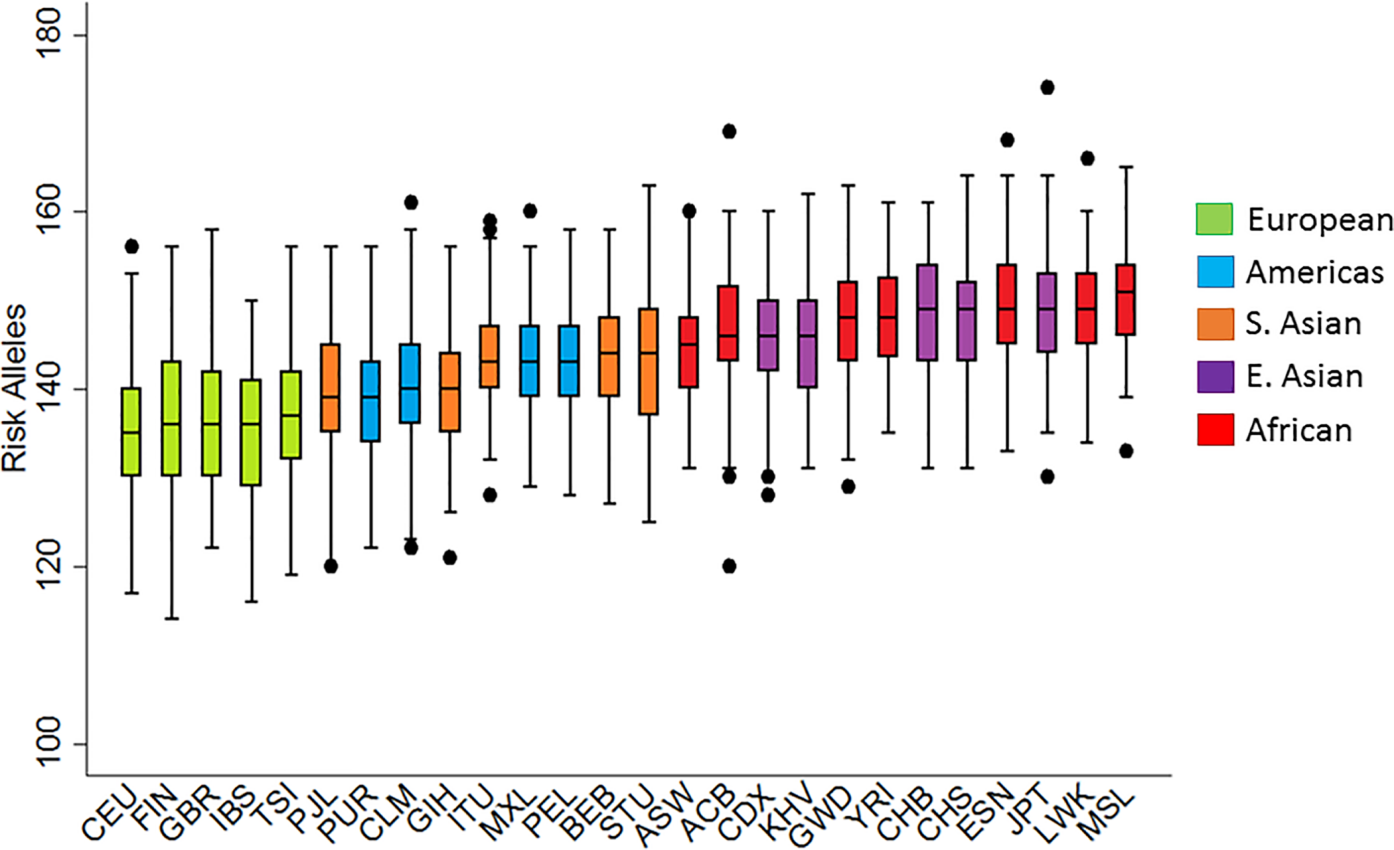
Distribution of fibroproliferative disease GRS in populations from 1000 Genomes. Results are sorted by median risk allele burden. Bars represent the 25^th^ and 75^th^ percentiles and are color coded by super-population (Green = EUR, Blue = AMR, Orange = SAS, Purple = EAS, Red = AFR).

This study sought to determine whether allele frequency differences at known fibroproliferative risk loci are consistent with evidence for selective pressure and may explain racial disparities associated with fibroproliferative diseases (and related quantitative phenotypes) through evaluating loci implicated by genome wide association studies (GWAS). To do this, a genetic risk score (GRS) utilizing single nucleotide polymorphisms (SNPs) from the GWAS catalog[19] was constructed for seven fibroproliferative diseases with increased prevalence (>2 fold on average) in African ancestry populations compared to European ancestry populations (Table 1). The total number of LD-pruned (r^2^<0.2) independent SNPs included in the GRS (excluding the *HLA* region) was 147 (S1 Table). This unweighted GRS was calculated in all 1000 Genomes[27] (a sample without phenotypic selection bias) samples as the number of risk-increasing alleles in each individual. The individual-level GRS burdens ranged from 114 to 174 risk alleles (Table 2, Fig 1, S2 Table). Overall, the burden of risk alleles was consistently higher in African-derived (AFR) populations (mean GRS = 148.07) than European-derived (EUR) populations (mean GRS = 136.03), which have the lowest overall GRS burden (Fig 1). The difference in the mean GRS values between the EUR and AFR populations was 12.04 risk alleles (t-test *p*-value = 1.75x10−123). This result became marginally more significant when limiting the AFR population to only continental African populations (difference in means = 12.69 risk alleles, *p*-value = 1.37x10^-125^).

**Table 2:** Summary statistics for fibroproliferative GRS among AFR and EUR populations from 1000 Genomes, and among BioVU samples.

Population	N	Mean GRS	Minimum GRS	Maximum GRS
**AFR**	**661**	**148.07**		
ACB	96	146.57	120	169
ASW	61	145.00	131	160
ESN	99	149.90	133	168
GWD	113	147.61	129	163
LWK	99	148.71	134	166
MSL	85	149.69	133	165
YRI	108	148.06	135	161
**BioVU AA (combined)**	**1382**	**145.11**		
BioVU AA cases	578	145.20	126.44	167.47
BioVU AA controls	804	145.05	117.41	164.25
**EUR**	**503**	**136.03**		
CEU	99	135.34	117	156
FIN	99	136.07	114	156
GBR	91	136.67	122	156
IBS	107	135.01	116	150
TSI	107	137.10	119	156
**BioVU EA (combined)**	**2359**	**136.34**		
BioVU EA cases	1195	136.13	113.20	162.18
BioVU EA controls	1164	136.55	113.94	161.10

GRS: Genetic risk score; AA: African American; EA: European American; CEU: Utah Residents (CEPH) with Northern and Western Ancestry; TSI: Toscani in Italia; FIN: Finnish in Finland; GBR: British in England and Scotland; IBS: Iberian Population in Spain; YRI: Yoruba in Ibadan, Nigeria; LWK: Luhya in Webuye, Kenya; GWD: Gambian in Western Divisions in the Gambia; MSL: Mende in Sierra Leone; ESN: Esan in Nigeria; ASW: Americans of African Ancestry in SW USA; ACB: African Caribbeans in Barbados

**Fig 2:**
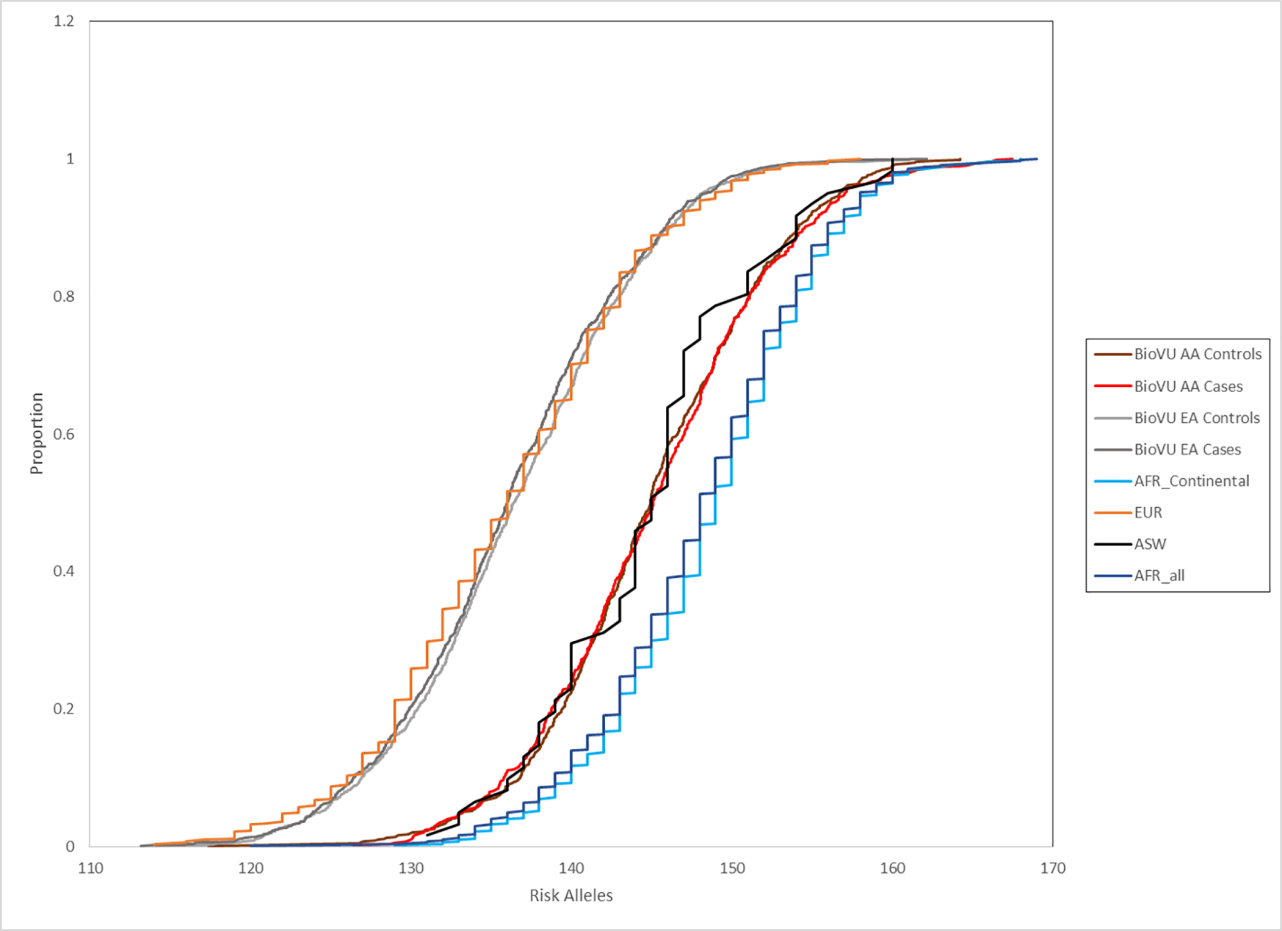
Cumulative distribution of GRS in 1000 Genomes AFR, EUR, ASW, and BioVU populations. *AFR includes only the continental African populations.

This was replicated in a larger independent population consisting of uterine fibroids cases and controls from BioVU, an electronic health record-linked DNA biorepository (Table 2, Fig 2), ascertained as part of another study[28]. In BioVU, the mean GRS in the African American samples was 145.11 (N = 1382; range: 117.41–167.47), while the mean in European American samples was 136.34 (N = 2359; range: 113.20–162.18). Cases and controls had similar means within racial groups, which were not significantly different from each other (African American controls mean = 145.05, African American cases mean = 145.20, t-test *p*-value = 0.69; European American controls mean = 136.55, European American cases mean = 136.13, t-test *p*-value = 0.16). The difference between African American and European American in this set was also highly significant (t-test *p*-value = 7.26x10^-237^), and remained so when evaluating controls only, as cases may be enriched for fibroproliferative alleles above those in the general population (t-test *p*-value = 6.38x10^-127^).

We further evaluated whether there are systematic differences in allele frequency at fibroproliferative disease risk alleles in the same direction as the prevalence disparity between African ancestry and European ancestry populations. Many approaches to evaluate selective pressure at multiple loci with small effects rely upon the proportionality assumption between disease effect sizes and allele frequency differences[26, 29]. However, those tests require that the disease of interest is itself under selective pressure, which is inconsistent with the hypothesis of selection in fibroproliferative diseases.

Therefore, evidence for selection was examined using the quantitative trait loci (QTL) sign test, which evaluates whether the number of variants at higher frequency in one population compared to the other deviates from neutrality under a binomial distribution[30]. This approach does not utilize phenotypic effect sizes, which is ideal for this study sample in which phenotypes are not observed and in this scenario where the effects of alleles on helminth infections have not been estimated. In this analysis, 85 of the 147 SNPs had risk alleles at higher frequency in African-derived populations than in European-derived populations (S3 Table), which is significant from the QTL sign test with a *p*-value of 0.011. Differences in risk allele frequency ranged from 0.006 to 0.73, with greater than 35% of the variants with an African-European frequency difference of 0.2 or larger. Thirty-three of the 53 variants (62%) with the largest (>0.2) differences were more common in African-derived populations (p-value = 0.022). The number of SNPs at higher frequency (and therefore the *p*-value for the sign test) did not change when limiting to samples from continental Africa. The sign test analysis also replicated in BioVU, with 84 of the 147 SNPs available in this dataset being more common in the African American samples compared to European American samples (*p*-value = 0.015).

## Conclusion

Overall, this analysis supports the possibility that selective pressure on an as-yet undetermined phenotype may have impacted genetic variants predisposing to seven fibroproliferative diseases. The observation made in the present study of higher genetic burden of fibroproliferative alleles was observed in both a population-based sample as well as within an independent sample ascertained from a hospital-based biobank. It is of note that nearly all of the genetic variants identified though GWAS (and thus included in the GRS) were implicated through studies of European or East Asian populations. This supports our conclusion, and identification of additional fibroproliferative risk variants that are more common in African populations will likely make the trends observed between these two population groups even more apparent.

## Methods

### Samples and genotypes

The discovery phase of these analyses utilized publically available genotype and population data from phase 3 of the 1000 Genomes Project was downloaded from ftp://ftp.1000genomes.ebi.ac.uk/vol1/ftp/[31]. Individual population and super-population assignments were the only non-genetic information evaluated.

For replication individuals with imaging-confirmed uterine fibroids and genome-wide genotype data were included from BioVU, Vanderbilt University Medical Center’s de-identified biorepository linked to electronic health records. The phenotyping algorithms used to identify case and control subjects have been previously published [28]. Briefly, this algorithm used a combination of demographic inclusion and exclusion criteria, International Classification of Diseases 9^th^ edition (ICD-9) diagnostic codes, Current Procedural Terminology (CPT) codes, and keywords exclusions from specific notes and reports of a participant in order to identify cases and controls. Cases required evidence of a fibroid diagnosis defined by either an ICD-9 code indicating the presence of fibroids or ICD and CPT codes indicating a history of fibroid treatment procedures (e.g. myomectomy or uterine artery embolization). An individual was included as a control if they had two imaging events on separate dates and did not have a fibroid diagnosis or history of fibroid treatment procedures. Excluded from controls were women without an intact uterus (e.g. having had a prior hysterectomy) based on CPT procedural codes and text mentions of hysterectomy. This study was reviewed and approved by the Vanderbilt University Institutional Review Board (#161378).

BioVU subjects were genotyped using both the Affymetrix BioBank array (European American and African American subjects) and the Axiom World array 2 (Affymetrix Inc., Santa Clara, CA) was additionally genotyped in the African Americans in order to attain better coverage for African-derived variants. Genotype quality control was performed separately for European American and African American datasets, including a 95% SNP and individual call rate threshold, removal of related individuals, gender checks, alignment of alleles to the genomic ‘+’ strand, and visualization of ancestry by principal components analysis using Eigenstrat software [32]. The genotype data were imputed to the 1000 Genomes phase 3 reference panel using SHAPEIT2 [33] for haplotype phasing and IMPUTE2 [34] for genotype imputation, with phasing and imputation performed separately for each dataset.

### Genetic risk score construction

A genetic risk score (GRS) utilizing single nucleotide polymorphisms (SNPs) from the GWAS catalog[19] was constructed for seven fibroproliferative diseases with increased prevalence (>2 fold on average) in African ancestry populations compared to European ancestry populations (Table 1). The total number of LD-pruned (r^2^<0.2) independent SNPs included in the GRS (excluding the *HLA* region) was 147 (S1 Table). This unweighted GRS was calculated in all 1000 Genomes[27] as the number of risk-increasing alleles in each individual. The GRS was also computed across the imputed BioVU data utilizing the dosage data to account for the number of risk-increasing alleles. Of the 147 variants evaluated, 70 were directly genotyped in the African American set, and 63 were genotyped in the European American set. Mean info scores among imputed SNPs were 0.979 and 0.971 in African American and European American sets, respectively.

## Supporting information

S1 Table List of SNPs included in the GRS. (PDF)

S2 Table Descriptive characteristics of the GRS by 1000 Genomes super-populations and BioVU samples. (PDF)

S3 Table Frequencies of GRS SNPs in 1000 Genomes super-populations and BioVU samples. AFR (cont.) represents continental AFR samples only (i.e. not including ACB and ASW populations).
(PDF)
