# Supplementary material for: Evidence of selection as a cause for racial disparities in fibroproliferative disease
Source: PLoS One. 2017 Aug 8;12(8):e0182791. doi: 10.1371/journal.pone.0182791 (PMC5549739; doi:10.1371/journal.pone.0182791)
Supplement: S1 Table — (PDF) [file pone.0182791.s001.pdf]

SNP	Disease	Chromosome	Position (hg19)	Risk Allele	Other Allele
rs880315	Hypertension	1	10796866	C	T
rs17367504	Hypertension	1	11862778	G	A
rs2275247	Systemic sclerosis	1	35908451	C	T
rs3753841	Glaucoma	1	103379918	G	A
rs12136063	Nephrosclerosis	1	110014170	A	G
rs10745332	Hypertension	1	113189053	A	G
rs17030613	Hypertension	1	113190807	C	A
rs267734	Nephrosclerosis	1	150951477	T	C
rs2049805	Nephrosclerosis	1	155194980	C	T
rs4656461	Glaucoma	1	165687205	G	A
rs3850625	Nephrosclerosis	1	201016296	G	A
rs873549	Keloid	1	222271767	C	T
rs2802729	Nephrosclerosis	1	243501763	A	C
rs807601	Nephrosclerosis	2	15793014	T	G
rs6431731	Nephrosclerosis	2	15863002	C	T
rs1260326	Nephrosclerosis	2	27730940	T	C
rs3213787	Glaucoma	2	45646824	A	G
rs7583877	Nephrosclerosis	2	100460654	C	T
rs11123170	Nephrosclerosis	2	113978940	G	C
rs17830558	Nephrosclerosis	2	160878364	T	G
rs16849225	Hypertension	2	164906820	C	T
rs1446468	Hypertension	2	164963486	T	C
rs4667594	Nephrosclerosis	2	170008506	A	T
rs4972593	Nephrosclerosis	2	174462854	A	T
rs7601754	Systemic sclerosis	2	191940451	A	G
rs2712184	Nephrosclerosis	2	217682779	A	C
rs6795744	Nephrosclerosis	3	13906850	A	G
rs820430	Hypertension	3	27548900	A	G
rs1717027	Hypertension	3	41987920	T	C
rs319690	Hypertension	3	47927484	C	T
rs9810888	Hypertension	3	53635595	G	T
rs13069000	Nephrosclerosis	3	66798950	G	C
rs1511412	Keloid	3	138713704	A	G
rs16833934	Hypertension	3	163737250	G	A
rs419076	Hypertension	3	169100886	T	C
rs16853722	Nephrosclerosis	3	169150632	C	T
rs9682041	Nephrosclerosis	3	170091902	C	T
rs6445055	Glaucoma	3	171992387	A	G
rs10513801	Nephrosclerosis	3	185822353	T	G
rs10937329	Nephrosclerosis	3	187713718	T	A
rs4619890	Glaucoma	4	7853160	G	A
rs3775948	Nephrosclerosis	4	9995182	C	G
rs871606	Hypertension	4	54799245	T	C
rs10032549	Nephrosclerosis	4	77398015	G	A
rs1902859	Hypertension	4	81157703	C	T
rs2725220	Nephrosclerosis	4	88959922	C	G

SNP	Disease	Chromosome	Position (hg19)	Risk Allele	Other Allele
rs228611	Nephrosclerosis	4	103561709	G	A
rs1173771	Hypertension	5	32815028	G	A
rs11959928	Nephrosclerosis	5	39397132	A	T
rs2233287	Systemic sclerosis	5	150440097	A	G
rs9313772	Hypertension	5	157804457	C	T
rs6420094	Nephrosclerosis	5	176817636	A	G
rs2745572	Glaucoma	6	1548369	G	A
rs11969985	Glaucoma	6	1922907	G	A
rs198846	Hypertension	6	26107463	G	A
rs7759001	Nephrosclerosis	6	27341409	G	A
rs3130573	Systemic sclerosis	6	31106268	G	A
rs3828890	Nephrosclerosis	6	31440669	G	C
rs805303	Hypertension	6	31616366	G	A
rs443198	Systemic sclerosis	6	32190406	G	A
rs9296015	Systemic sclerosis	6	32218989	A	G
rs9272729	Nephrosclerosis	6	32609594	A	G
rs881858	Nephrosclerosis	6	43806609	A	G
rs13209747	Hypertension	6	127115454	T	C
rs1936800	Nephrosclerosis	6	127436064	T	C
rs17080102	Hypertension	6	151004770	G	C
rs2279463	Nephrosclerosis	6	160668389	A	G
rs316009	Nephrosclerosis	6	160675764	T	C
rs10277115	Nephrosclerosis	7	1285195	T	A
rs59072263	Glaucoma	7	8152067	G	T
rs17428471	Hypertension	7	27337867	T	G
rs3750082	Nephrosclerosis	7	32919927	T	A
rs6465825	Nephrosclerosis	7	77416439	T	C
rs7801190	Hypertension	7	100458093	C	G
rs17477177	Hypertension	7	106411858	T	C
rs10258482	Glaucoma	7	116150095	A	C
rs7805747	Nephrosclerosis	7	151407801	A	G
rs6459680	Nephrosclerosis	7	156258568	T	G
rs1015213	Glaucoma	8	52887541	T	C
rs284491	Glaucoma	8	105958633	T	C
rs2071518	Hypertension	8	120435812	T	C
rs7827545	Hypertension	8	135566567	T	C
rs4744712	Nephrosclerosis	9	71434707	C	A
rs2487032	Glaucoma	9	107703934	A	G
rs8176743	Glaucoma	9	136131415	T	C
rs1044261	Nephrosclerosis	10	1065710	C	T
rs10795433	Nephrosclerosis	10	16969923	A	C
rs4373814	Hypertension	10	18419972	C	G
rs11014166	Hypertension	10	18708798	A	T
rs10994860	Nephrosclerosis	10	52645424	T	C
rs1530440	Hypertension	10	63524591	T	C
rs9663362	Hypertension	10	95895177	G	C

SNP	Disease	Chromosome	Position (hg19)	Risk Allele	Other Allele
rs12416687	Hypertension	10	104629011	T	C
rs7913069	Uterine fibroids	10	105714399	T	C
rs2782980	Hypertension	10	115781527	C	T
rs2280543	Uterine fibroids	11	203788	C	T
rs163160	Nephrosclerosis	11	2789955	A	G
rs7129220	Hypertension	11	10350538	A	G
rs381815	Hypertension	11	16902268	C	T
rs11024102	Glaucoma	11	17008605	C	T
rs10767873	Nephrosclerosis	11	30768678	C	T
rs12419342	Glaucoma	11	47468545	C	T
rs747782	Glaucoma	11	47940925	C	T
rs479777	Sarcoidosis	11	64107477	T	C
rs504915	Nephrosclerosis	11	64464085	T	A
rs4014195	Nephrosclerosis	11	65506822	C	G
rs633185	Hypertension	11	100593538	G	C
rs11222084	Hypertension	11	130273230	T	A
rs1106766	Nephrosclerosis	12	57809456	T	C
rs626277	Nephrosclerosis	13	72347696	C	A
rs17536527	Nephrosclerosis	15	45719187	C	G
rs491567	Nephrosclerosis	15	53946593	C	A
rs8032158	Keloid	15	56194877	C	T
rs3825942	Glaucoma	15	74219582	A	G
rs893818	Glaucoma	15	74229195	A	G
rs6495122	Hypertension	15	75125645	A	C
rs7172677	Systemic sclerosis	15	75424593	A	C
rs1394125	Nephrosclerosis	15	76158983	A	G
rs2521501	Hypertension	15	91437388	T	A
rs12437854	Nephrosclerosis	15	94141833	G	T
rs13329952	Nephrosclerosis	16	20366507	T	C
rs11864909	Nephrosclerosis	16	20400839	C	T
rs889472	Nephrosclerosis	16	79645989	C	A
rs12711490	Systemic sclerosis	16	85973028	T	C
rs164748	Nephrosclerosis	16	89708292	C	G
rs11656696	Glaucoma	17	10033679	A	C
rs2453580	Nephrosclerosis	17	19438321	T	C
rs7208487	Nephrosclerosis	17	37543449	T	G
rs12946454	Hypertension	17	43208121	T	A
rs17608766	Hypertension	17	45013271	T	C
rs16948048	Hypertension	17	47440466	G	A
rs11868441	Nephrosclerosis	17	59239221	G	A
rs8068318	Nephrosclerosis	17	59483766	C	T
rs7227483	Nephrosclerosis	18	43187130	G	A
rs8091180	Nephrosclerosis	18	77164243	A	G
rs12460876	Nephrosclerosis	19	33356891	C	T
rs11666497	Nephrosclerosis	19	38464262	C	T
rs3116139	Glaucoma	19	51879034	C	T

SNP	Disease	Chromosome	Position (hg19)	Risk Allele	Other Allele
rs1327235	Hypertension	20	10969030	G	A
rs6088580	Nephrosclerosis	20	33285053	C	G
rs6066043	Nephrosclerosis	20	45288453	A	G
rs17216707	Nephrosclerosis	20	52732362	T	C
rs6026584	Nephrosclerosis	20	57469073	C	T
rs6015450	Hypertension	20	57751117	G	A
rs35934224	Glaucoma	22	19872645	C	T
rs2239785	Nephrosclerosis	22	36661330	G	A
rs12484776	Uterine fibroids	22	40652873	G	A
