# Supplementary material for: Evidence of selection as a cause for racial disparities in fibroproliferative disease
Source: PLoS One. 2017 Aug 8;12(8):e0182791. doi: 10.1371/journal.pone.0182791 (PMC5549739; doi:10.1371/journal.pone.0182791)
Supplement: S2 Table — (PDF) [file pone.0182791.s002.pdf]

SNP	AFR	AFR (cont.)	ACB + ASW	EUR	AMR	SAS	EAS	EA Controls	EA Cases	AA Controls	AA Cases
rs6431731	0.002	0.000	0.010	0.036	0.014	0.005	0.001	0.057	0.049	0.011	0.017
rs1511412	0.005	0.001	0.016	0.111	0.065	0.104	0.036	0.107	0.103	0.022	0.019
rs6066043	0.006	0.002	0.019	0.141	0.221	0.272	0.546	0.139	0.153	0.048	0.040
rs11024102	0.017	0.001	0.067	0.258	0.268	0.329	0.371	0.294	0.276	0.062	0.060
rs3116139	0.018	0.008	0.051	0.288	0.297	0.359	0.284	0.263	0.263	0.068	0.078
rs16853722	0.027	0.023	0.041	0.125	0.094	0.176	0.296	0.127	0.111	0.055	0.053
rs1530440	0.036	0.037	0.035	0.190	0.232	0.154	0.194	0.198	0.173	0.072	0.074
rs7129220	0.052	0.056	0.041	0.129	0.068	0.044	0.001	0.114	0.106	0.079	0.079
rs9272729	0.053	0.049	0.067	0.106	0.118	0.076	0.052	0.121	0.125	0.071	0.072
rs17030613	0.064	0.069	0.045	0.235	0.278	0.210	0.442	0.195	0.199	0.090	0.076
rs12484776	0.065	0.057	0.092	0.194	0.434	0.301	0.287	0.210	0.205	0.099	0.113
rs12946454	0.067	0.058	0.096	0.283	0.197	0.305	0.224	0.248	0.251	0.093	0.094
rs11868441	0.068	0.035	0.175	0.797	0.754	0.886	0.756	0.812	0.803	0.208	0.194
rs1106766	0.082	0.076	0.099	0.194	0.310	0.101	0.079	0.247	0.230	0.103	0.108
rs1902859	0.089	0.076	0.131	0.310	0.311	0.259	0.366	0.320	0.310	0.118	0.132
rs13069000	0.090	0.088	0.096	0.184	0.133	0.060	0.196	0.196	0.179	0.098	0.129
rs1260326	0.094	0.089	0.108	0.411	0.362	0.200	0.481	0.395	0.422	0.146	0.133
rs316009	0.101	0.103	0.096	0.109	0.069	0.134	0.041	0.108	0.100	0.091	0.088
rs17367504	0.110	0.102	0.137	0.139	0.078	0.188	0.123	0.167	0.152	0.101	0.131
rs10277115	0.117	0.100	0.169	0.755	0.533	0.679	0.308	0.757	0.756	0.238	0.252
rs13209747	0.120	0.106	0.162	0.451	0.362	0.417	0.489	0.428	0.440	0.171	0.179
rs7759001	0.123	0.112	0.156	0.235	0.231	0.260	0.413	0.236	0.224	0.145	0.129
rs8091180	0.125	0.111	0.169	0.597	0.601	0.457	0.784	0.561	0.574	0.194	0.178
rs2745572	0.150	0.144	0.169	0.364	0.382	0.484	0.493	0.339	0.360	0.179	0.179
rs17428471	0.154	0.165	0.118	0.069	0.081	0.026	0.037	0.089	0.078	0.115	0.122
rs7601754	0.154	0.124	0.252	0.819	0.787	0.880	0.855	0.823	0.817	0.279	0.257
rs880315	0.165	0.159	0.185	0.357	0.513	0.398	0.636	0.341	0.348	0.195	0.174
rs7913069	0.166	0.174	0.143	0.038	0.117	0.069	0.085	0.021	0.019	0.156	0.138
rs8176743	0.169	0.176	0.150	0.084	0.048	0.233	0.194	0.062	0.074	0.158	0.166
rs893818	0.178	0.180	0.172	0.319	0.307	0.309	0.522	0.337	0.322	0.210	0.203
rs12437854	0.179	0.187	0.156	0.079	0.153	0.127	0.215	0.065	0.063	0.158	0.143
rs873549	0.181	0.179	0.188	0.283	0.369	0.434	0.352	0.284	0.287	0.200	0.170
rs633185	0.196	0.199	0.185	0.277	0.421	0.500	0.484	0.271	0.294	0.235	0.222

SNP	AFR	AFR (cont.)	ACB + ASW	EUR	AMR	SAS	EAS	EA Controls	EA Cases	AA Controls	AA Cases
rs11656696	0.201	0.199	0.207	0.414	0.337	0.249	0.489	0.416	0.421	0.241	0.247
rs6015450	0.205	0.202	0.213	0.127	0.059	0.051	0.000	0.124	0.124	0.187	0.187
rs11222084	0.209	0.209	0.207	0.330	0.330	0.217	0.022	0.366	0.352	0.225	0.221
rs2233287	0.213	0.218	0.194	0.082	0.091	0.047	0.003	0.082	0.083	0.182	0.172
rs10994860	0.222	0.216	0.242	0.202	0.110	0.200	0.047	0.174	0.172	0.230	0.206
rs2521501	0.223	0.218	0.239	0.319	0.183	0.232	0.095	0.304	0.299	0.237	0.226
rs10032549	0.225	0.205	0.287	0.516	0.484	0.337	0.273	0.536	0.528	0.272	0.260
rs6795744	0.231	0.222	0.258	0.158	0.133	0.037	0.081	0.154	0.152	0.223	0.202
rs9663362	0.232	0.225	0.255	0.528	0.369	0.488	0.300	0.523	0.531	0.283	0.298
rs4656461	0.256	0.261	0.242	0.141	0.135	0.043	0.009	0.125	0.115	0.218	0.208
rs9296015	0.266	0.266	0.268	0.220	0.236	0.194	0.299	0.197	0.176	0.240	0.221
rs12136063	0.269	0.256	0.309	0.689	0.818	0.714	0.957	0.714	0.704	0.351	0.341
rs12460876	0.270	0.255	0.319	0.423	0.375	0.445	0.639	0.387	0.403	0.285	0.273
rs11123170	0.278	0.285	0.258	0.394	0.367	0.471	0.333	0.373	0.383	0.312	0.292
rs11959928	0.287	0.289	0.280	0.451	0.395	0.377	0.174	0.432	0.441	0.305	0.334
rs9682041	0.291	0.308	0.236	0.121	0.111	0.163	0.060	0.121	0.121	0.260	0.253
rs7172677	0.293	0.290	0.303	0.301	0.246	0.267	0.201	0.269	0.266	0.298	0.280
rs3828890	0.296	0.295	0.299	0.124	0.140	0.055	0.032	0.115	0.101	0.315	0.306
rs6088580	0.311	0.302	0.341	0.465	0.314	0.401	0.408	0.441	0.456	0.342	0.349
rs10795433	0.313	0.295	0.373	0.836	0.591	0.739	0.594	0.841	0.833	0.392	0.385
rs8032158	0.315	0.325	0.283	0.280	0.300	0.432	0.304	0.317	0.324	0.313	0.313
rs7805747	0.325	0.336	0.287	0.288	0.173	0.071	0.003	0.272	0.269	0.313	0.327
rs3750082	0.330	0.323	0.350	0.635	0.700	0.497	0.656	0.630	0.658	0.410	0.390
rs881858	0.338	0.332	0.357	0.695	0.761	0.756	0.773	0.708	0.686	0.361	0.363
rs1015213	0.338	0.365	0.252	0.101	0.055	0.108	0.014	0.093	0.084	0.268	0.298
rs4972593	0.349	0.352	0.338	0.134	0.104	0.403	0.214	0.164	0.160	0.310	0.298
rs17536527	0.350	0.357	0.325	0.480	0.644	0.598	0.909	0.424	0.443	0.353	0.342
rs1394125	0.350	0.350	0.350	0.342	0.233	0.229	0.083	0.357	0.362	0.340	0.342
rs805303	0.357	0.341	0.408	0.633	0.572	0.698	0.631	0.624	0.638	0.410	0.420
rs6445055	0.362	0.380	0.306	0.174	0.278	0.205	0.339	0.161	0.162	0.332	0.348
rs7827545	0.370	0.352	0.427	0.335	0.398	0.460	0.673	0.320	0.311	0.352	0.362
rs10258482	0.398	0.415	0.344	0.258	0.208	0.191	0.009	0.272	0.288	0.357	0.350
rs16948048	0.408	0.434	0.325	0.371	0.244	0.140	0.211	0.363	0.386	0.402	0.373

SNP	AFR	AFR (cont.)	ACB + ASW	EUR	AMR	SAS	EAS	EA Controls	EA Cases	AA Controls	AA Cases
rs3825942	0.410	0.419	0.382	0.177	0.169	0.268	0.140	0.148	0.166	0.368	0.376
rs747782	0.424	0.437	0.385	0.193	0.170	0.163	0.259	0.204	0.202	0.386	0.403
rs2712184	0.442	0.427	0.490	0.572	0.627	0.457	0.533	0.567	0.591	0.488	0.486
rs443198	0.471	0.478	0.446	0.364	0.378	0.502	0.562	0.381	0.378	0.475	0.486
rs6459680	0.482	0.468	0.528	0.754	0.741	0.723	0.628	0.753	0.752	0.558	0.549
rs3130573	0.488	0.492	0.475	0.347	0.317	0.324	0.184	0.351	0.344	0.442	0.458
rs2802729	0.490	0.506	0.440	0.445	0.396	0.571	0.319	0.463	0.464	0.487	0.484
rs6465825	0.493	0.494	0.490	0.574	0.689	0.638	0.780	0.595	0.599	0.507	0.522
rs1327235	0.494	0.489	0.510	0.462	0.264	0.490	0.542	0.482	0.479	0.503	0.510
rs1936800	0.535	0.547	0.497	0.480	0.510	0.484	0.492	0.510	0.525	0.517	0.539
rs2782980	0.538	0.536	0.545	0.697	0.777	0.806	0.862	0.711	0.722	0.563	0.547
rs419076	0.550	0.558	0.526	0.482	0.369	0.473	0.151	0.470	0.492	0.528	0.522
rs9810888	0.551	0.559	0.529	0.489	0.405	0.409	0.417	0.520	0.499	0.546	0.544
rs7583877	0.568	0.572	0.554	0.269	0.401	0.226	0.365	0.316	0.304	0.540	0.560
rs2071518	0.592	0.589	0.599	0.225	0.189	0.308	0.161	0.262	0.270	0.506	0.539
rs2453580	0.600	0.598	0.605	0.593	0.732	0.833	0.787	0.601	0.603	0.613	0.597
rs4744712	0.601	0.605	0.586	0.623	0.751	0.688	0.545	0.587	0.601	0.575	0.599
rs16833934	0.604	0.612	0.576	0.272	0.179	0.151	0.216	0.261	0.267	0.540	0.534
rs4373814	0.613	0.619	0.592	0.466	0.514	0.431	0.504	0.425	0.424	0.557	0.585
rs491567	0.617	0.617	0.615	0.203	0.404	0.276	0.531	0.226	0.221	0.567	0.546
rs2049805	0.617	0.599	0.675	0.523	0.689	0.547	0.757	0.519	0.508	0.633	0.629
rs7227483	0.620	0.622	0.615	0.745	0.741	0.805	0.845	0.735	0.714	0.635	0.612
rs4667594	0.623	0.631	0.599	0.512	0.647	0.488	0.135	0.541	0.543	0.603	0.574
rs319690	0.627	0.639	0.589	0.300	0.404	0.347	0.285	0.307	0.304	0.608	0.592
rs12419342	0.653	0.685	0.551	0.302	0.313	0.162	0.341	0.298	0.292	0.567	0.564
rs13329952	0.657	0.657	0.656	0.784	0.744	0.681	0.914	0.803	0.810	0.652	0.658
rs7208487	0.659	0.648	0.694	0.838	0.764	0.921	0.762	0.836	0.840	0.655	0.669
rs3775948	0.663	0.661	0.672	0.755	0.575	0.684	0.581	0.761	0.740	0.657	0.677
rs889472	0.676	0.688	0.640	0.376	0.517	0.532	0.595	0.392	0.390	0.633	0.652
rs626277	0.689	0.706	0.634	0.417	0.587	0.629	0.876	0.407	0.382	0.649	0.652
rs35934224	0.691	0.686	0.707	0.846	0.856	0.814	0.967	0.841	0.839	0.725	0.721
rs2239785	0.694	0.716	0.621	0.219	0.160	0.162	0.204	0.217	0.195	0.636	0.639
rs1717027	0.704	0.717	0.662	0.191	0.186	0.173	0.149	0.174	0.172	0.632	0.639

SNP	AFR	AFR (cont.)	ACB + ASW	EUR	AMR	SAS	EAS	EA Controls	EA Cases	AA Controls	AA Cases
rs284491	0.719	0.740	0.650	0.341	0.416	0.272	0.406	0.328	0.341	0.647	0.673
rs2275247	0.720	0.730	0.688	0.050	0.367	0.241	0.766	0.038	0.037	0.633	0.638
rs11969985	0.741	0.739	0.745	0.851	0.833	0.859	0.822	0.848	0.862	0.763	0.738
rs7801190	0.750	0.742	0.774	0.946	0.937	0.956	0.998	0.957	0.954	0.785	0.777
rs59072263	0.756	0.749	0.777	0.858	0.911	0.880	0.908	0.849	0.853	0.782	0.775
rs6495122	0.775	0.796	0.710	0.470	0.512	0.873	0.818	0.429	0.432	0.726	0.708
rs10745332	0.783	0.789	0.764	0.756	0.865	0.879	0.811	0.741	0.752	0.784	0.774
rs871606	0.792	0.796	0.780	0.863	0.859	0.664	0.801	0.890	0.898	0.791	0.812
rs1173771	0.795	0.799	0.783	0.581	0.591	0.613	0.661	0.619	0.607	0.767	0.775
rs2725220	0.801	0.817	0.752	0.486	0.395	0.405	0.241	0.490	0.481	0.744	0.736
rs8068318	0.803	0.818	0.755	0.272	0.579	0.529	0.632	0.277	0.284	0.688	0.728
rs381815	0.810	0.812	0.806	0.705	0.710	0.872	0.842	0.742	0.719	0.808	0.796
rs228611	0.812	0.819	0.787	0.518	0.683	0.403	0.514	0.529	0.525	0.750	0.739
rs2279463	0.814	0.806	0.841	0.872	0.934	0.916	0.918	0.870	0.868	0.810	0.815
rs6420094	0.815	0.821	0.793	0.668	0.721	0.698	0.787	0.682	0.674	0.804	0.776
rs6026584	0.818	0.829	0.780	0.665	0.719	0.752	0.748	0.666	0.658	0.783	0.789
rs4014195	0.819	0.812	0.841	0.649	0.785	0.654	0.763	0.659	0.672	0.792	0.792
rs807601	0.822	0.832	0.787	0.323	0.434	0.561	0.783	0.356	0.353	0.739	0.757
rs2487032	0.833	0.844	0.796	0.498	0.484	0.560	0.492	0.502	0.496	0.761	0.776
rs9313772	0.846	0.858	0.806	0.664	0.650	0.629	0.830	0.621	0.622	0.813	0.816
rs4619890	0.859	0.875	0.806	0.466	0.597	0.619	0.704	0.476	0.477	0.824	0.824
rs10937329	0.866	0.873	0.844	0.681	0.736	0.813	0.606	0.700	0.684	0.822	0.837
rs11014166	0.867	0.866	0.869	0.678	0.784	0.708	0.880	0.666	0.669	0.845	0.854
rs17080102	0.880	0.876	0.892	0.930	0.806	0.831	0.920	0.933	0.934	0.900	0.897
rs198846	0.881	0.880	0.885	0.822	0.865	0.926	0.965	0.835	0.838	0.891	0.884
rs12416687	0.887	0.896	0.857	0.746	0.814	0.925	0.821	0.751	0.748	0.853	0.856
rs479777	0.914	0.923	0.885	0.660	0.634	0.778	0.834	0.693	0.647	0.879	0.855
rs16849225	0.914	0.923	0.885	0.775	0.800	0.712	0.595	0.766	0.770	0.891	0.882
rs11864909	0.927	0.932	0.911	0.706	0.718	0.690	0.866	0.723	0.727	0.889	0.882
rs10767873	0.929	0.941	0.892	0.502	0.588	0.743	0.672	0.529	0.549	0.864	0.872
rs12711490	0.930	0.940	0.898	0.805	0.831	0.944	0.926	0.781	0.769	0.899	0.893
rs504915	0.935	0.959	0.857	0.287	0.585	0.389	0.781	0.313	0.313	0.847	0.818
rs2280543	0.935	0.933	0.943	0.967	0.961	0.895	0.845	0.952	0.956	0.935	0.937

SNP	AFR	AFR (cont.)	ACB + ASW	EUR	AMR	SAS	EAS	EA Controls	EA Cases	AA Controls	AA Cases
rs164748	0.937	0.952	0.885	0.523	0.672	0.805	0.984	0.549	0.554	0.886	0.882
rs163160	0.938	0.942	0.927	0.821	0.813	0.742	0.792	0.823	0.819	0.907	0.933
rs11666497	0.940	0.940	0.940	0.805	0.857	0.794	0.890	0.800	0.810	0.924	0.929
rs17477177	0.941	0.947	0.920	0.777	0.843	0.701	0.864	0.801	0.800	0.924	0.924
rs3753841	0.951	0.980	0.857	0.388	0.245	0.414	0.297	0.369	0.396	0.820	0.868
rs17830558	0.959	0.985	0.876	0.466	0.713	0.746	0.656	0.445	0.468	0.887	0.882
rs1446468	0.961	0.978	0.908	0.488	0.667	0.596	0.462	0.474	0.470	0.864	0.883
rs1044261	0.962	0.964	0.955	0.934	0.957	0.993	1.000	0.917	0.929	0.960	0.954
rs17216707	0.965	0.974	0.936	0.773	0.648	0.785	0.957	0.817	0.804	0.918	0.931
rs820430	0.980	0.993	0.940	0.610	0.627	0.623	0.329	0.618	0.598	0.920	0.911
rs10513801	0.988	0.994	0.968	0.862	0.921	0.875	0.937	0.872	0.870	0.958	0.959
rs17608766	0.990	0.999	0.962	0.826	0.942	0.958	0.999	0.876	0.872	0.979	0.977
rs3213787	0.990	0.988	0.997	0.967	0.863	0.894	0.864	0.961	0.959	0.989	0.988
rs267734	0.993	0.997	0.981	0.806	0.906	0.946	0.974	0.799	0.798	0.960	0.954
rs3850625	0.995	1.000	0.978	0.882	0.912	0.802	0.960	0.878	0.889	0.983	0.981
