# Supplementary material for: Evidence of selection as a cause for racial disparities in fibroproliferative disease
Source: PLoS One. 2017 Aug 8;12(8):e0182791. doi: 10.1371/journal.pone.0182791 (PMC5549739; doi:10.1371/journal.pone.0182791)
Supplement: S3 Table — AFR (cont.) represents continental AFR samples only (i.e. not including ACB and ASW populations). (PDF) [file pone.0182791.s003.pdf]

	AFR (cont.)	AFR	ACB + ASW	EAS	SAS	AMR	EUR	EA Controls	EA Cases	AA Control	AA Cases
N Samples	504	661	157	504	489	347	503	1164	1195	804	578
min	0.000	0.002	0.010	0.000	0.005	0.014	0.036	0.021	0.019	0.011	0.017
mean	0.506	0.504	0.497	0.502	0.483	0.480	0.463	0.464	0.463	0.497	0.497
median	0.494	0.493	0.497	0.504	0.471	0.434	0.451	0.428	0.440	0.506	0.522
max	1.000	0.995	0.997	1.000	0.993	0.961	0.967	0.961	0.959	0.989	0.988
sd	0.321	0.315	0.300	0.312	0.279	0.270	0.258	0.260	0.261	0.291	0.293
